# Exploring different methods to evaluate the impact of basic income interventions: a systematic review

**DOI:** 10.1186/s12939-021-01479-2

**Published:** 2021-06-16

**Authors:** Andrew D. Pinto, Melissa Perri, Cheryl L. Pedersen, Tatiana Aratangy, Ayu Pinky Hapsari, Stephen W. Hwang

**Affiliations:** 1 MAP Centre for Urban Health Solutions, Li Ka Shing Knowledge Institute, Unity Health Toronto, Toronto, Canada; 2 Department of Family and Community Medicine, St. Michael’s Hospital, Toronto, Canada; 3 Department of Family and Community Medicine, Faculty of Medicine, University of Toronto, Toronto, Canada; 4 Dalla Lana School of Public Health, University of Toronto, Toronto, Canada; 5 Division of General Internal Medicine, Department of Medicine, University of Toronto, Toronto, Canada

**Keywords:** Basic income, Income inequality, Social determinants of health, Methodology, Health, Equity.

## Abstract

**Background:**

Persistent income inequality, the increase in precarious employment, the inadequacy of many welfare systems, and economic impact of the COVID-19 pandemic have increased interest in Basic Income (BI) interventions. Ensuring that social interventions, such as BI, are evaluated appropriately is key to ensuring their overall effectiveness. This systematic review therefore aims to report on available methods and domains of assessment, which have been used to evaluate BI interventions. These findings will assist in informing future program and research development and implementation.

**Methods:**

Studies were identified through systematic searches of the indexed and grey literature (Databases included: Scopus, Embase, Medline, CINAHL, Web of Science, ProQuest databases, EBSCOhost Research Databases, and PsycINFO), hand-searching reference lists of included studies, and recommendations from experts. Citations were independently reviewed by two study team members. We included studies that reported on methods used to evaluate the impact of BI, incorporated primary data from an observational or experimental study, or were a protocol for a future BI study. We extracted information on the BI intervention, context and evaluation method.

**Results:**

86 eligible articles reported on 10 distinct BI interventions from the last six decades. Workforce participation was the most common outcome of interest among BI evaluations in the 1960–1980 era. During the 2000s, studies of BI expanded to include outcomes related to health, educational attainment, housing and other key facets of life impacted by individuals’ income. Many BI interventions were tested in randomized controlled trials with data collected through surveys at multiple time points.

**Conclusions:**

Over the last two decades, the assessment of the impact of BI interventions has evolved to include a wide array of outcomes. This shift in evaluation outcomes reflects the current hypothesis that investing in BI can result in lower spending on health and social care. Methods of evaluation ranged but emphasized the use of randomization, surveys, and existing data sources (i.e., administrative data). Our findings can inform future BI intervention studies and interventions by providing an overview of how previous BI interventions have been evaluated and commenting on the effectiveness of these methods.

**Registration:**

This systematic review was registered with PROSPERO (CRD 42016051218).

**Supplementary Information:**

The online version contains supplementary material available at 10.1186/s12939-021-01479-2.

## Background

Income inequality has risen in many high-income countries since the 1970s, resulting in many individuals not benefiting from the increase in societal wealth creation. Comparatively, poverty rates have remained high in many low- and middle-income countries [1–3]. Current welfare policies around the world do not adequately protect members of society, particularly during times of financial crisis [4]. Even in high income countries such as Canada, the United States, and the United Kingdom, many argue that the existing welfare policies are discriminatory and perpetuate system-level barriers for marginalized populations. Some examples of these barriers include the complicated and time-consuming bureaucracy processes to access the services, extensive policing on benefit eligibility, and the prioritization of monetary values that neglect the client’s morale and quality of life [4–6].

One proposal to improve income security and reduce the onerous nature of welfare is a basic income (BI), also referred to as Universal Basic Income, Guaranteed Minimum Income, Basic Income Guarantee, negative income tax (NIT) or a demogrant [7, 8]. A BI is given to all who meet the income eligibility criteria, is simple to administer, and is unconditional in nature (i.e. does not require recipients to seek work) [9]. The idea of providing all members of society a BI dates back to ancient times and represents a payment from the government to ensure recipients achieve a minimum income level [10–12].

The idea of a BI gained popularity in the 1960s and 1970s, inspiring field experiments in the United States and Canada [10]. However, the neoliberal era of the 1980s and 1990s was associated with reductions in government spending and a decline in financial supports for those who were unemployed or disabled [13–15]. Within the past decade, growing income insecurity, precarious employment, and concern around displacement of manual labour by automation and artificial intelligence have sparked a renewed interest in BI [16, 17]. The dialogue around BI further gained momentum during the COVID-19 pandemic in 2020, that resulted in a rapid loss of employment, the rise of income inequality, and substantial economic uncertainty [18–20]. To reduce COVID-19 transmission, many countries enacted lockdowns that led to business closures and worsening economic conditions [21, 22]. Several high-income countries, such as Canada and New Zealand, have moved to provide some form of emergency income support to assist their citizens who are financially impacted by the pandemic [23, 24]. However, many argue that these temporary benefits will not be able to sustainably address the widespread and long-term impact of COVID-19 [25, 26]. Instead, a permanent BI can be explored as a potential solution to reduce this gap and bring positive impacts to various aspects of people’s lives, including their health and overall quality of lives [27]. Some proponents of BI also suggested BI as a potential next step in the evolution of social welfare [28]. Further, the similarities between BI and some policy measures implemented during the pandemic have presented a unique opportunity to study the effect of BI policies on different life domains [26].

One of the most compelling reasons for investing in BI is that it may produce cost savings through reduced health and social service costs [29, 30]. This rationale is supported by numerous studies that found a correlation between low income, worse health outcomes, and higher use of the health care system [31, 32]. In the Canadian BI field experiment (1974–1979), research suggested that receiving BI was associated with reduced hospitalizations, physician visits and some improvements in mental health [10].

Academics have long been interested in studying the impact of BI interventions on various social, health and labour-specific outcomes. The COVID-19 pandemic has also spurred further interest in the exploration of BI as part of post COVID-19 economic recovery plans and as a potential long-term solution to reduce the poverty rate [33]. Relevant experiments have occurred worldwide before the pandemic, in locations such as Namibia (2008–2010), India (2011–2012), Kenya (2011–2013) and Finland (2017–2018). However, no synthesis has been done that consolidates methods of evaluation across BI specific outcomes. Our objective was to search peer-reviewed and grey literature to identify and examine methods used to evaluate the impact of BI interventions. Specifically, this review provides a repository of BI evaluation methods, including study design, data collection methods, and outcome domains that can be adopted by researchers and policy makers who wish to implement a BI intervention. This review also identifies outcome domains that were overlooked in existing BI evaluations. Finally, heterogeneity in the approaches to data analysis and important considerations around BI implementation are described.

## Methods

### Search strategy and selection criteria

Relevant articles published on or before January 30, 2020 were searched from the following databases: Scopus, Embase, Medline, CINAHL, Web of Science, ProQuest databases, EBSCOhost Research Databases, and PsycINFO. The start year of the publication was not specified to filter the search results. To supplement searches of the indexed literature, grey literature sources were searched in consultation with an Information Specialist **(**Additional File 1). The following search terms were used in all of the databases: “basic income”, “guaranteed annual income”, “guaranteed minimum income”, “minimum income”, “negative income tax”, “optimal income transfer*”, mincome, demogrant*, “citizen* income”, or “universal income”. We also searched Google, Google Scholar, Open Grey and Campbell Library along with websites that focused on BI interventions. Additional studies were identified through expert contacts and the reference list of included studies.

Studies had to meet all three of the following inclusion criteria: 1) The article had to report on a BI intervention or program, which provided guaranteed income unconditionally to low-income individuals. Studies describing selective interventions, such as the Bolsa Familia or universal interventions in regions such as the Netherlands, were excluded due to pre-defined conditional requirements to gaining income [34]. 2) The study had to report on the methods used to evaluate the impact of BI interventions. Reporting on the methods used to implement a basic income scheme was not sufficient for inclusion. 3) The article had to report primary data from an observational study (e.g., case-control, cohort), experimental study (e.g., randomized controlled trial or RCT), or protocol for future research. Reviews, opinions, commentaries and editorials, or literature that specifically focused on a subset of the population (e.g., children, seniors) were excluded.

### Data extraction, synthesis and presentation

Articles that were identified through the search were uploaded to two systematic literature review software: Covidence and DistillerSR. Each citation was reviewed independently by two team members. Citations that were potentially relevant were retrieved for full-text review by two team members. The authors were contacted for papers that could not be retrieved. The search was not limited by language. Studies that were not available in English were reviewed by an individual fluent in the appropriate language and/or translated into English. Any disagreements on whether to include or exclude an article in each stage of screening were resolved by the project coordinator and the principal investigator (ADP).

Data from included papers was extracted by one team member and then confirmed by a second team member. The extraction form was created using MS Excel and included the following variables: citation; purpose; methods used (including type of study, type of BI implemented, dates, data collection methods, evaluation methods, data analysis methods); location; sample; participant demographics; outcome domains; and results. Data was subsequently synthesized by examining common patterns surrounding the methods of data collection used to evaluate the impact of BI interventions along with common outcomes analyzed by each study. Furthermore, we examined the commonalities in the data analysis methods across the included studies and made note of any external data sources that were triangulated with data collected during the BI interventions. In addition, any considerations around implementation of a BI intervention discussed by the authors were also noted and analyzed to identify the common themes. As this systematic review is focused on methodology rather than outcomes, when a large number of outcomes were reported in one resource (e.g., a book or final report about an experiment) the main outcome and health outcomes were prioritized.

The quality of 53 articles that included primary data analysis was assessed using the Joanna Briggs Institute (JBI) critical appraisal tools [35]. The JBI tools were selected because of the heterogeneity in the study designs captured in our review and JBI offers a comprehensive selection of pragmatic checklists that address different types of study. Twenty-three articles were assessed using the checklist for RCTs, and the same number of articles were assessed using the economic evaluation checklist. Four articles were simultaneously evaluated using checklists for RCTs and economic evaluations, given their adoption of both designs [36–39]. Checklist for case series was used to critically appraise 2 articles [40, 41], and another article was assessed using the checklist for quasi-experimental studies [42].

## Results

**Fig. 1:**
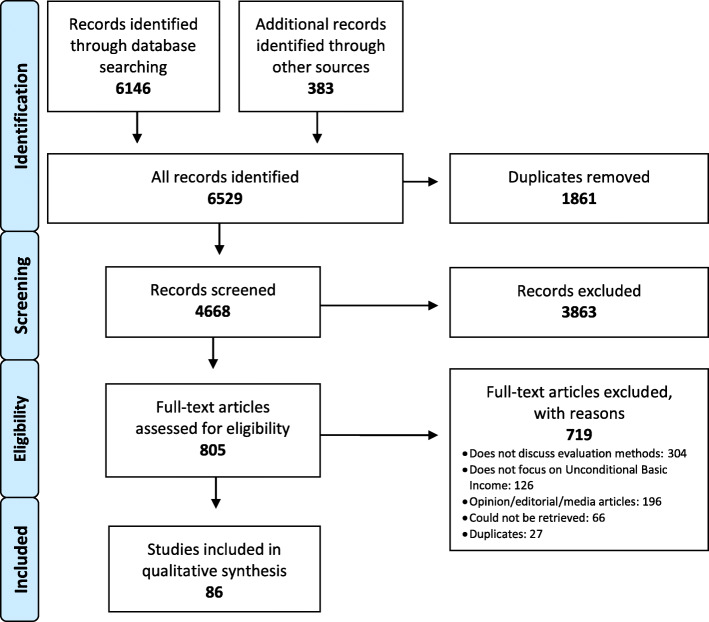
Selection Process of Articles

Our search identified 6146 citations by database and 383 citations through grey literature searches, expert contacts and search of reference lists. After removing duplicate publications, 4668 citations remained for screening. Following abstract and title review, 805 full-text citations were assessed for eligibility. The most common reason for exclusion was a failure to discuss methods to evaluate an unconditional BI experiment (*n* = 304). During full-text review, 27 additional duplicates were identified. Sixty-six articles could not be retrieved, the majority were published more than 30 years ago. Only two studies from the grey literature search met inclusion criteria. A total of 86 citations met the eligibility criteria for inclusion (Fig. 1). Of these, fifty-three articles conducted analysis of the primary data and were critically appraised (Additional File 2)**.**

**Fig. 2:**
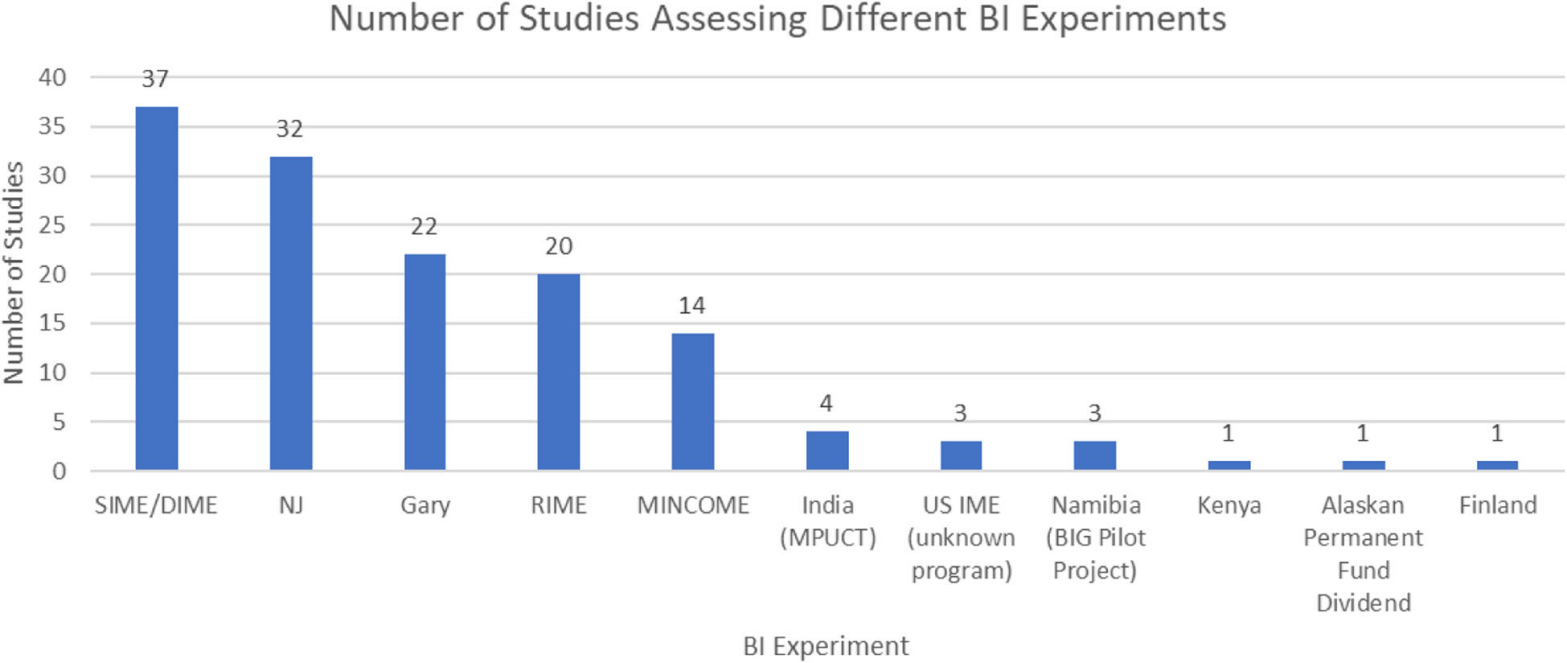
Number of Studies Assessing Different BI Experiments ^a^. ^a^ The total number of studies exceeded 86 because several studies included data from more than one BI experiment

Of the 86 articles included, 63 were based on studies in the United States, 9 in Canada, and five were focused on studies in both the United States and Canada. The remaining articles were about the Madhya Pradesh Unconditional Cash Transfers Project (MPUCT) in India (*n* = 4), the BIG Pilot Project in Namibia (*n* = 3), and BI programs in Kenya (*n* = 1) and Finland (n = 1). Most of the American and Canadian studies (*n* = 77) were based on 5 experiments that involved BI programs that utilized a NIT model. NIT, or negative income tax, is a system that allows the state to pay benefits to people whose income falls below a certain threshold of tax liability, while people whose income exceeds the threshold pay tax to the state. As their income increases, the amount of benefits received by eligible individuals also decreases [43]. One US study assessed a citizen’s dividend model that was implemented in Alaska. Under this system, all Alaskan residents are entitled to a yearly dividend payment from the Alaska Permanent Fund, which is funded by the state’s oil revenues [42]. The Indian, Namibian, Kenyan, and Finland BI programs involved unconditional cash transfers (UCTs). Figure 2 presents the number of studies that emerged from each of the BI programs captured in this review.

### Details of BI studies

Ten basic income experiments and programs were identified in this review, with four of them being carried out in the United States from the 1960s–70s: the New Jersey Income Maintenance Experiment (NJ), The Rural Income Maintenance Experiment (RIME), the Gary Income Maintenance Experiment (Gary), and the Seattle-Denver Income Maintenance Experiment (SIME/DIME). These American income maintenance experiments were the first Randomized Controlled Trials (RCT) conducted in a community setting rather than a clinical setting. The main purpose for all of the experiments was to measure the labour supply response to BI guarantee (i.e. would BI result in a decline of work effort). In total, eight BI experiments included in this review were designed as RCTs, with only the BIG project in Namibia and the Alaska Permanent Dividend Fund in the US adopting observational design (Table 1). The sample sizes in the BI experiments that implemented an RCT design were relatively large, ranging from 809 participants in the RIME experiment to 11,688 participants in the MPUCT (Table 1). The BIG project in Namibia involved 398 individuals, while the study on the Alaska Permanent Dividend Fund observed data from about 48 million individuals (Table 1).

**Table 1:** Basic Income Experiments and Programs

Basic Income Experiment/ Program	Location	Study Design	Years	Sample #^a^	Sample Description	Survey Timeline	Income Data Collection	Other Data Sources
New Jersey Income Maintenance Experiment (NJ)	Trenton, Paterson, Passaic, Jersey City, New Jersey; Scranton, Pennsylvania, USA	Randomized Controlled Trial	1968–1972	1357 households (725 intervention; 632 control [44])	Adult males 18–58 and at least one other family member	Pre-enrollment Interview, Baseline Interview, Twelve Quarterly Interviews, a follow-up interview 3 months after the last payment was made.	Income Data Forms;Pay stubs;Social Security aggregate data Periodic Audit Forms	- Schools, hospitals, public organizations offering services to the poor, and other relevant institutions and organizations - Family composition reporting: monthly for experimental and every 4 months for controls
Rural Income Maintenance Experiment (RIME)	Duplin County, Iowa; Calhoun and Pocahontas Counties, North Carolina, USA	Randomized Controlled Trial	1970–1972	809 families (372 intervention; 437 control [44])	Adults 18–58 including either two-parent or single parent households headed by females with at least one other family member; rural areas	Baseline Interview, 12 quarterly Interviews, follow-up interview 3 months after the last payment	Periodic Audit Forms	- Schools, hospitals, public organizations offering services to the poor, and other relevant institutions and organizations - Family composition reporting: monthly for experimental and every 4 months for controls
Gary Income Maintenance Experiment (Gary)	Gary, Indiana, USA	Randomized Controlled Trial	1971–1974	1799 families (1028 intervention; 771 control [44])	Black adults 18–58 including single parent families especially headed by females and at least one other family member	Baseline interview and eight interviews about every 4 months	Monthly Income Report FormsPeriodic Audit Forms	- Family composition reporting: monthly for experimental and every 4 months for controls
Seattle/Denver Income Maintenance Experiment (SIME/DIME)	Seattle, Washington; Denver, Colorado, USA	Randomized Controlled Trial	1970–1976	4800 families (2747 intervention; 2053 control [44])	Adults 18–58 with at least one other family member	Baseline interview and interviews about every 4 months	Monthly Income Report Forms Periodic Audit Forms	- Family composition reporting: monthly for experimental and every 4 months for controls
Manitoba Guaranteed Annual Income Experiment (MINCOME)	Winnipeg, Manitoba, CanadaSaturation Site: Rural community of Dauphin and a number of small rural communities.	Randomized Controlled Trial	1974–1979	> 1300 families (575 intervention; 612 control [44]	Adults 18–58 with at least one other family member; Institutionalized, retired, or disabled were excluded from Winnipeg. Elderly and disabled not excluded from Dauphin (saturation site).	Baseline interview and interviews 3 times per year	None listed	- The Manitoba Population Health Research Data Repository database - Use of aggregate data from the Department of Education
Alaska Permanent Dividend Fund	Alaska, USA	Observational: Differences in Difference	1977–2015	48,686, 169	All residents of Alaska and all residents of control states that have been matched with Alaska on employment to population ratio and the population share working part-time	Monthly surveys, with some questions asked at fourth and eighth month of the survey (Integrated Public Use Microdata Series – Current Population Surveys (CPS) provided by the Minnesota Population Center)	None listed	- CPS Merged Outgoing Rotation Groups (MORG) provided by the National Bureau of Economic Research (NBER) for number of hours worked
Namibia Basic Income Grant Pilot Project	Otjivero-Omitara, Namibia	Observational: cohort	2008–2010	398 individuals [40]	All individuals under 60 years	Baseline, 6-month, 11-month surveys	None listed	- Key informant interviews: e.g., local nurse, police chief, local leaders and shop keepers - Case studies including pictures
Madhya Pradesh Unconditional Cash Transfer (MPUCT)	Rural Villages, Indore District; Rural Tribal Villages, Indore District, Madhya Pradesh, India	Modified Randomized Controlled Trial	2011–2012	11,688 individuals; 2034 households (8 villages randomly assigned to intervention; 12 villages to control [45])	Universal: all individuals within the villages	Baseline survey, interim evaluation survey, final evaluation survey, post-final evaluation Survey	None listed	- Case Studies including structured interviews - Community level surveys - Interviews with key respondents - Tracking of children’s weight for age - Tracking of children’s attendance and school performance
Give Directly Unconditional Cash Transfer Program	Rural Rarieda Region, Kenya	Two-level cluster-Randomized Controlled Trial	2011–2013	1440 households (503 intervention; 432 “pure” control in control villages; 505 “spillover” control in intervention villages [36])	Individuals living in a house with a thatch roof	Baseline and endline surveys	None listed	- Saliva samples to measure cortisol levels - Height, weight, and upper-arm circumference of children under five years - Interviews with village dwellers
Finland Basic Income Experiment	Mixed Rural and Urban Areas in Finland	Randomized Control Trial	2017–2018	175,000 (for the registry data) and 1633 (for survey)	Individuals aged 25–58 receiving unemployment benefits from Kela (Finnish social insurance institution)	Baseline survey and one interim survey nearing the end of the intervention	None listed	- Administrative data on employment - Qualitative interviews (results not reported in the preliminary report)

^a^Sample size is often not consistently reported. The sample size is referenced throughout

The NJ Experiment ran from 1968 to 1972 and was proposed to measure the labour supply response of urban males aged 18–58 [46]. To address the gap in knowledge with respect to BI impacts on labour supply in rural communities, RIME was initiated [47]. RIME took place in various locations across the United States, included single parent female headed households and was conducted from 1970 to 1972 [48]. Gary was an NIT experiment that was carried out from 1971 to 1974 and involved urban families who were mostly black and headed by females, which distinguished it from the other BI interventions in the US that involved a high proportion of white families [49, 50]. Finally, the SIME/DIME study had a considerably larger sample size than the other American experiments and was the only experiment with a treatment duration longer than 3 years, spanning from 1970 to 1976 [51, 52]. The sample assignment for each study utilized the Conlisk-Watts Assignment Model, which was developed to scientifically optimize placement of participants within the treatment groups based on characteristics relevant to the outcomes being analyzed (Table 1) [44].

The Manitoba Income Maintenance Experiment (MINCOME) was also conducted from 1974 to 1979 in urban Winnipeg and rural Dauphin, Manitoba. This experiment was designed as an RCT and was the only one to include a saturation site (Dauphin, Manitoba), which presented an opportunity to look at the effects a BI would have on the community as a whole. The focus of this experiment was also on labour supply, similar to the American experiments. Unfortunately, the majority of the data collected during the study was never analyzed due to budgetary constraints (Table 1) [10].

Conducted from 2008 to 2010, the Namibian BIG Pilot Project was purported to be the first universal UCT program in the world and was designed as an observational cohort experiment [40]. A monthly BI grant was provided to approximately 1000 participants, of whom 398 were followed with surveys every 6 months. The main purposes of the pilot were to reduce poverty and improve social behaviours and the local economy. The intervention has been reported as having a large positive effect on the main outcomes as well as health outcomes [40, 41].

The Madhya Pradesh Unconditional Cash Transfer Project (MPUCT) was conducted in India from 2011 to 2012 and included 11,688 individuals [45]. This experiment adopted the RCT study design and a cluster sampling method. In this experiment, villages, rather than individuals, were randomly selected for the intervention or control sites. All participants living within a selected experimental village received the BI payments for 1 year. The main purpose of the experiment was to lift people out of poverty (Table 1) [53].

An UCT program was also conducted in Kenya from 2011 to 2013 with 1440 individuals. This project used an RCT design and involved multiple treatment arms ranging from a one-time transfer payment to nine monthly installments. Household recipients were also randomized (in addition to households being randomly selected to participate). The main purpose of the experiment included measuring work effort response, behavioural effects and psychological well-being (Table 1) [36].

In January 2017, Finland introduced a partial BI experiment that followed two thousand recipients aged 25–58 years for 2 years. Following the RCT principle, participants were sampled randomly from the Finnish population who received regular unemployment benefit without any regional or other emphasis. Similar to the other BI experiments, their main purpose was to investigate whether a BI could provide stronger incentive for workforce participation than the current social security system. The preliminary findings of the experiment were published in 2019 and utilized both administrative and survey data (Table 1) [54].

Lastly, an observational study completed in the US examined the impact of a permanent fund dividend provided to Alaskan residents on the state’s labour market. This cash transfer was introduced in 1977 and has continued until the present. The study included in this review used population survey and census data from 1977 to 2015. The impact of the fund was measured by evaluating the change in employment and part-time jobs, change in labour force participation, and change in worked hours (Table 1) [42].

### Methods used in BI studies

The articles included in the review evaluated the impacts of BI intervention on seven major domains. About half of the papers (*n* = 40) focused on outcomes relating to labour supply or workforce participation. The second most common domain to be evaluated was health outcomes (*n* = 11), including fertility, hospitalization rates, nutrition, and birth weights, followed by income level and living conditions (*n* = 10). Family stability, particularly marital dissolution, also became the focus of nine articles based on the four major BI experiments in the US, namely NJ, RIME, Gary, and SIME/DIME. Several articles (*n* = 7) also examined elements that can affect the implementation and analysis of a BI experiment, henceforth described as the methods domain, such as potential underreporting of employment status and income level, stigma that may hinder participation in a BI experiment and other social welfare, and the potential understatement of the effects of BI intervention due to the temporary nature of the experiment [55–57]. The two other major domains that were assessed include education-related outcomes (*n* = 6) and asset ownership [3]. A few other articles also examined the impact of BI intervention on awareness of social services, risk-taking in labour market and rate of migration [58–61]. Details of the main outcomes evaluated in each article are described in Table 2.

**Table 2:** Data Extraction Outcomes

No	Authors, Year	Basic Income Experiment	Type of Basic Income	Country	Main Outcome	Main Outcome Results
1	Ashenfelter, 1990 [74]	SIME/DIME	NIT	USA	Labour supply	Incentive effects on labour supply would have a real effect on the transfer costs of such a program
2	Baumol, 1974 [73]	US IMEs (not specified)	NIT	USA	Housing consumption behaviour	Increase in home ownership
3	Bawden, 1970 [48]	NJ, RIME	NIT	USA	Work incentive	None
4	Beck et al., 2015 [45]	India	UCT	India	Illness or injury in the household and vaccination coverage	Less minor illness or injury in the household found in the intervention group, but no effect on vaccination coverage and serious illness and injuries
5	SEWA Bharat, 2014 [53]	India	UCT	India	Basic living conditions including sanitation, drinking water, energy sources	Basic living improved including sanitation, energy sources
6	Bishop, 1980 [69]	NJ, RIME, Gary, SIME/DIME	NIT	USA	Marital dissolution rates	Rates of marital dissolution were higher
7	Brodkin and Kaufman, 2000 [87]	NJ, SIME/DIME	NIT	USA	Labour supply	Labour supply showed very little evidence of a work disincentive
8	Burtless, 1986 [88]	NJ, RIME, Gary, SIME/DIME	NIT	USA	Work hours and earnings	Work hours and earnings are decreased among NIT recipients across all four experiments
9	Burtless and Greenberg, 1981 [68]	SIME/DIME	NIT	USA	Work hours	The estimated reduction in hours is larger when the analysis focuses on those below the breakeven level
10	Burtless and Greenberg, 1982 [89]	SIME/DIME	NIT	USA	Labour supply	Reduction in labor supply was larger in the 5-year experimental sample than the 3-year one
11	Burtless and Hausman, 1978 [90]	Gary	NIT	USA	Labour supply	Labour supply is largely unaffected by NIT, but it may be reduced by poor health and aging
12	Byrne, 1973 [91]	NJ, Gary	NIT	USA	Mother’s work disincentives with day-care costs	Mothers have a disincentive to work when faced with day-care costs
13	Cain and Wissoker, 1990 [72]	SIME/DIME	NIT	USA	Marital Stability when accounting for potential bias from different durations of experiments	Impact of NIT on marital breakups decline with the length of the programs
14	Cain et al., 1974 [92]	NJ	NIT	USA	Labour supply of married women	Disincentives with labor-force participation for white wives
15	Calnitsky, 2016 [55]	MINCOME	NIT	Canada	Community experience	- Motives to participate in MINCOME are related to money/assistance - Social stigma experienced by MINCOME recipients was lower than welfare recipients
16	Choudhry and Arvin, 2001 [93]	MINCOME	NIT	Canada	Family income level and marital dissolution	Increase in marital dissolution with lower family income level
17	Choudhry and Hum, 1995 [94]	MINCOME	NIT	Canada	Income level and marital dissolution	NIT payments did not encourage splits by lowering the financial costs of marital disruption
18	Connor, Rodgers, and Priest, 1999 [44]	NJ, RIME, Gary, SIME/DIME, MINCOME	NIT	USA and Canada	Marital stability, nutrition, education	No consistent effects on marital stability, nutrition or education
19	Curry, 1981 [95]	NJ	NIT	USA	Work effort	Work effort did not decline
20	Davala et al., 2015 [76]	India	UCT	India	Debt and Borrowing	Debt and borrowing reduced
21	Dickinson and Watts, 1975 [85]	NJ, RIME	NIT	USA	Uses of the Data: labor supply response	Labor supply response was focus of experimental design
22	Elesh and Lefcowitz, 1977 [96]	NJ	NIT	USA	Health and health care utilization	No effect observed on health and health care utilization.
23	Forget, 2011 [10]	MINCOME	NIT	Canada	Hospitalization rates	Hospitalization rates fell
24	Forget, 2013 [64]	MINCOME	NIT	Canada	Hospital separations	Hospital separations declined
25	Forget, 2010 [97]	NJ, RIME, Gary, SIME/DIME, MINCOME	NIT	USA and Canada	Work effort	US: reduction in work effort
26	Forget et al., 2013 [98]	MINCOME	NIT	Canada	Hospitalization rates	Hospitalization rates fell
27	Greenberg and Halsey, 1983 [56]	SIME/DIME	NIT	USA	Underreporting of employment and quarterly earnings	Higher degree of underreporting of employment status and earning by intervention recipients compared to that by the control group
28	Greenberg, Moffitt, Friedmann, 1981 [65]	Gary	NIT	USA	Underreporting and work effort	Underreporting work effort was substantial
29	Groeneveld et al., 1980 [70]	SIME/DIME	NIT	USA	Marital dissolution	SIME/DIME increased the rate of marital dissolution
30	Haarmann, 2008 [40]	BIG Pilot Project (Namibia)	UCT	Namibia	Nutrition and child development	Reduced food shortages
31	Haarmann et al., 2009 [41]	Namibia	UCT	Namibia	Poverty Rates	Poverty rates decrease
32	Haushofer and Shapiro, 2013 [36]	Kenya	UCT	Kenya	AssetsConsumption	Increased Asset Values
33	Heffernan, 1977 [60]	RIME	NIT	USA	Awareness of social services	No effect on awareness of social services
34	Hollister, 1974 [99]	NJ	NIT	USA	Total family hours and total family earnings	Significant response to the experimental treatment by Whites and Spanish-speakers for total family hours and total family earnings.
35	Hum and Choudry, 1992 [100]	MINCOME	NIT	Canada	Family income level and marital dissolution	Social roles expected of each partner, not family income, determines family stability
36	Hum and Simpson, 1991 [101]	MINCOME	NIT	Canada	Labour Supply Response of Families and Individuals	Negative effect on work hours for single female heads
37	Hum and Simpson, 1993 [102]	NJ, RIME, Gary, SIME/DIME, MINCOME	NIT	USA and Canada	Labor supply response	Insignificant changes in work behaviour
38	Huston, 1999 [103]	NJ, RIME, Gary, SIME/DIME	NIT	USA	Children’s educational outcomes	Mixed effects on children’s school performance and attendance, achievement and aspirations, high school completion, educational attainment, employment.
39	Johnson, 1980 [61]	US IMEs (not specified)	NIT	USA	Risk taking in labour market	Risk-taking rises with income
40	Jones and Marinescu, 2018 [42]	Alaskan Permanent Fund Dividend	UCI	USA	Evolution in labour market	No change in employment within Alaska pre- and post-dividend payment, and no difference in labour market evolution between Alaska and other states
41	Kaluzny, 1979 [49]	NJ, Gary	NIT	USA	Home ownership	Increases in homeownership
42	Kangas et al., 2019 [104]	Finland	UCI	Finland	Work effort	No difference in average days in open employment, but slight increase in self-employment rate and their associated earnings
43	Keeley et al., 1978 [105]	SIME/DIME	NIT	USA	Labour supply	Income effects are negative for wives and female heads; Substitution effects are positive
44	Keeley et al., 1978 [106]	SIME/DIME	NIT	USA	Labour supply effects and costs of alternative negative income tax programs	Labor-supply response and program costs vary widely with the support level and tax rate
45	Keeley and Robins, 1979 [107]	SIME/DIME	NIT	USA	Work disincentives	SIME/DIME reduced hours of work
46	Keeley, 1980 [58]	SIME/DIME	NIT	USA	Migration	Increased rate of mobility for white married males and females
47	Keeley, 1980 [108]	SIME/DIME	NIT	USA	Fertility	NIT negatively effects married whites’ and positively effects married Chicanos’ fertility
48	Keeley, 1980 [59]	SIME/DIME	NIT	USA	Rate of migration	Increased rate of migration
49	Keeley, 1987 [109]	SIME/DIME	NIT	USA	Marital Dissolution	Positive effect on marital dissolution/divorce rates
50	Kehrer and Wolin, 1979 [63]	Gary	NITTwo guarantee levels and two tax rates	USA	Birth weight	No difference in birth weight
51	Kerachsky, 1977 [110]	RIME	NIT	USA	Farm family labor supply	Changes in guarantee produce a pattern of negative effects on labor supply
52	Kershaw, 1972 [111]	NJ	NIT	USA	Earnings	No evidence indicating a significant decline in weekly earnings
53	Kershaw and Fair, 1976 [84]	NJ	NIT	USA	Withdrawal from Work	No substantial withdrawal from work
54	Levine et al., 2005 [112]	NJ, RIME, Gary, SIME/DIME	NIT	USA	Labour supply	Reduction of work effort
55	Maynard, 1977 [66]	RIME	NIT	USA	School performance	Improvements in school performance; Increases in educational attainment.
56	Maynard and Murnane, 1979 [113]	Gary	NIT	USA	School performance	Increase in average reading achievement for grades 4–6
57	McDonald and Stephenson, 1979 [114]	Gary	NIT	USA	School enrollment	Being males increased rate of school enrollment and reduced labor force participation
58	Metcalf, 1973 [57]	US IMEs (not specified)	NIT	USA	Temporary NIT results extended to permanent NIT results	A temporary experiment will 1) understate the income effect and 2) overstate the gross and compensated price effects of the NIT.
59	Moffitt, 1979 [115]	Gary	NIT	USA	Employment status and unconditional hours worked	Labor supply reductions for husbands and female heads, but not for wives
60	Moffitt, 1981 [71]	NJ, RIME, Gary, SIME/DIME	NIT	USA	Weekly work hours	Reduced weekly work hours
61	Moffit and Kehrer, 1981 [75]	NJ, RIME, Gary, SIME/DIME	NIT	USA	Weekly work hours	Reduced weekly work hours
62	Munnell et al., 1987 [116]	NJ, RIME, Gary, SIME/DIME	NIT	USA	The effect of the NIT Treatments on Work Effort and Labour Supply	Reduction in work effort for most subsamples
63	Murray and Pateman, 2012 [67]	MINCOME	NIT	Canada	High school continuation	Adolescent males did continue in high school longer
64	Neuberg, 1988 [62]	SIME/DIME	NIT	USA	Distortion in reporting hours worked	Distortion revealed in reporting hours worked
65	Nicholson and Wright 1977 [37]	NJ	NIT	USA	Participants’ understanding of the NIT	The NIT was not well understood by experimental participants
66	O’Connor and Madden, 1979 [117]	RIME	NIT	USA	Diet	Little, if any, influence on the quality of the diets of the Iowa families; evidence of a beneficial effect on the quality of the diets of the North Carolina families.
67	Osterkamp, 2013 [78]	BIG Coalition (Namibia)	Universal Unconditional Cash Transfer	Namibia	Poverty reduction	Poverty was substantially reduced
68	Robins, 1980 [38]	SIME/DIME	NIT	USA	The effect of the NIT treatments on the labor supply of youth	The effect of the NIT Treatments on the Labor Supply of Youth
69	Robins, 1985 [118]	NJ, RIME, Gary, SIME/DIME	NIT	USA	Labour supply	Labour supply reduced
70	Robins, Tuma, and Yaeger, 1980 [119]	SIME/DIME	NIT	USA	Rates of leaving and entering employment	Higher rate of leaving employment and lower rate of entering employment
71	Robins and West, 1986 [120]	SIME/DIME	NIT	USA	Impacts of accounting differences between study attriters and non-attriters on employment and earnings estimates	Weighting techniques that considered differences between attriters and non-attriters did not have significant impact on the employment and earnings estimates
72	Ross, 1970 [121]	NJ	NIT	USA	Work Disincentive Effects	No evidence of work disincentive response
73	Rossi and Rosenbaum, 1983 [77]	NJ	NIT	USA	Work effort	Work effort declined slightly
74	Salkind and Haskins, 1982 [50]	NJ, RIME, Gary, SIME/DIME	NIT	USA	Fertility, nutrition, birth weight	Lower fertility; quality of nutrition increased; Fewer low birth weight babies
75	Skidmore, 1974 [46]	NJ	NIT	USA	None	None (paper only describes the type of data that are available from the experiment)
76	Spiegelman and Yaeger, 1980 [83]	SIME/DIME	NIT	USA	Labor supply	Husbands and single-family heads left employment more readily
77	Standing, 2015 [122]	India	UCT	India	Debt	Less likely to increase debt and more likely to reduce it
78	Stephens, 2007 [123]	SIME/DIME	NIT	USA	Work hours for men in dual-headed households	Hours of work reduction for men in dual-headed households was greater for the 5-year experiment than the 3-year one
79	Watts, 1969 [124]	NJ	NIT	USA	Participation	Almost all of those who have been invited to participate in the payments program have chosen to do so
80	Weiss, Hall, and Dong, 1980 [125]	SIME/DIME	NIT	USA	Schooling investment	Increase in schooling investment
81	West, 1980 [126]	SIME/DIME	NIT	USA	Wage rates	Little basis to indicate any effect on wage rates
82	West, 1980 [127]	SIME/DIME	NIT	USA	Work effort among non-heads of families	Reduction in work effort among non-heads
83	Widerquist, 2005 [51]	NJ, RIME, Gary, SIME/DIME, MINCOME	NIT	USA and Canada	Work-effort reduction	Work-effort reduced
84	Widerquist, 2013 [128]	NJ, RIME, Gary, SIME/DIME, MINCOME	NIT	USA and Canada	Work Disincentive Results	Longer periods of nonemployment or return to work
85	Wright and Wright, 1975 [129]	NJ	NIT	USA	Labour force participation	No difference in incentive to work
86	Wright, 1975 [39]	NJ	NIT	USA	Work disincentive effects	No statistically significant work disincentive effects

**Fig. 3:**
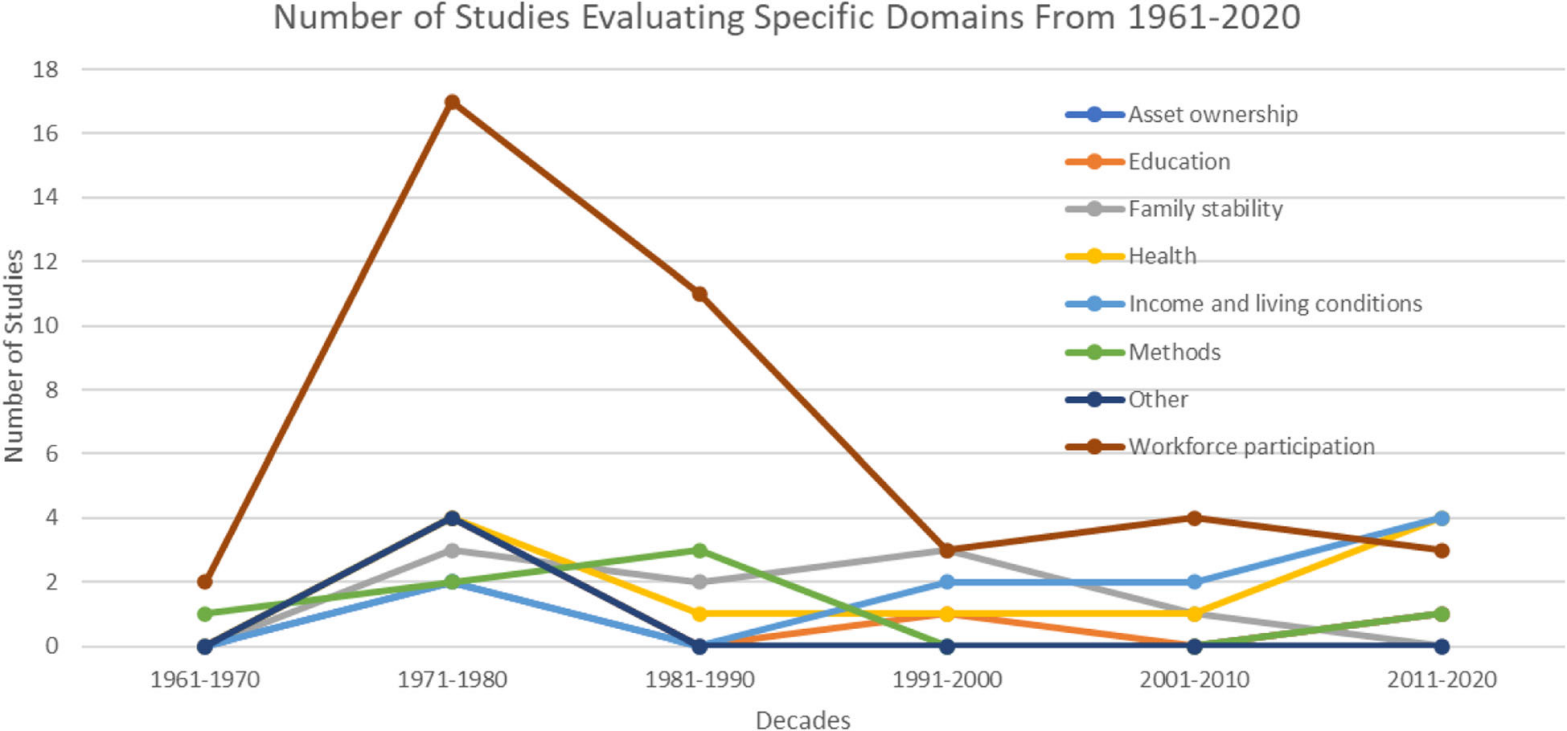
Number of Citations Evaluating Specific Domains from 1961-2020^a^. ^a^ This figure includes five studies that evaluated impact of BI on two domains and were counted twice as a result. The count in this figure also excludes one study that did not assess a specific domain of outcome

Throughout the past six decades, workforce participation consistently became the most evaluated domains of BI intervention, with up to 17 articles drawing information from labour data taken during the BI experiments in the 1970’s (Fig. 3). Some study interest on health-related outcomes was also shown during the 1970’s, before declining in the next three decades and picking up again in 2010’s. Family stability was also consistently evaluated between the 1970’s and the 1990’s, but research interest in this domain seemed to decrease in the 2000’s. Conversely, the impacts of BI on income level and living conditions only began to gain interest in the last two decades.

The data collection process for each of the experimental studies involved either interviews at baseline and periodic points in time throughout the intervention period or the use of surveys. The NJ had the greatest quantity of surveys, including a pre-enrollment, baseline, twelve quarterly interviews and a follow-up interview. The other US-based experiments followed a similar structure, most including a baseline interview, 8–12 quarterly interviews and follow-up interviews. The MINCOME experiment ended data collection after the third round of interviews. The MPUCT experiment was unique in their utilization of evaluation-based surveys that were administered at interim, final and post-final time points.

Each experiment utilized a wide array of data sources to gather information on the participants’ eligibility to receive benefits, their demographics, and the outcome of interests. There was a concern about underreporting of income during the BI experiments using the NIT model, particularly among participants in the treatment arm, given the amount of benefit that they were eligible for would decrease as their income increased [62]. To address this concern, several BI experiments involved multiple forms of income substantiation to determine the amount of benefits that the participants were able to claim. Compared to other US-based interventions, the NJ held the most robust income data collection method, as information was gathered using income data forms, pay stubs, social security aggregate data and periodic audit forms. In contrast, Gary and RIME only utilized self-reported income information and periodic audit forms (Table 1). Additional data sources that were included during the BI experiments in the US included family composition reporting and social- and health-related organizations.

Nine articles complemented the data that was collected during the duration of the BI experiment with other data sources. In most cases, this involved administrative data that provided further information on the specific domain in question. For example, a study examining the impact of NIT on birth weight utilized data taken from the Indiana’s certificate of birth [63]. Other studies also triangulated survey or interview data with administrative data related to health (*n* = 2), school performance and resources (n = 2), and employment and income level (*n* = 3), while another study combined data taken from multiple population-based surveys to examine employment rate (*n* = 1) [10, 42, 54, 56, 64–67].

No specific income-based data sources were listed for MINCOME; however, information was gathered using the Manitoba Population Health Research Data Repository Database along with information from the Department of Education. Similarly, no income-specific data source was reported in the preliminary findings of the Finnish experiment, although administrative data capturing indicators of work effort response was supplemented with analysis on survey data to measure a number of health outcomes. Comparably, the MPUCT, Namibian and Kenyan projects did not mention income-specific data sources utilized. Rather, each experiment included interviews with key informants such as local stakeholders, local leaders, shopkeepers, village dwellers, as well as health care providers and police chiefs [36, 40, 53]. Both MPUCT and the Give Directly Unconditional Cash Transfer program collected measurements on children’s weight. A unique aspect of the Namibian BIG project included its incorporation of case studies relating to outcomes [40]. The use of these case studies provides valuable context on the poverty in the region and the living conditions of the participants before the BIG intervention, the participants’ expectations for the intervention, and how the intervention compared to their expectations.

The evaluation approach of the Alaskan Permanent Fund Dividend is different from those employed in other studies. Jones and Marinescu [42] used the employment to population ratio and the share of the population working part-time when comparing the evolution of labour market outcomes in Alaska before and after the introduction of the dividend payments. The observed data were divided into pre-dividend years, covering July 1979 up to the introduction of dividend payment in June 1982, and post-dividend years from July 1982 to June 2015. All outcomes of the study were measured through analysis of existing survey data, namely the Current Population Survey.

There were 64 studies in this review that either analyzed data collected during the BI experiments or proposed a specific approach of analysis, with most of them adopting some form of multi-variate statistical modelling (*n* = 60) in their analyses, while other studies conducted bivariate (*n* = 6) or univariate analysis (*n* = 9). Economic modelling and simple regressions were the two most used statistical models, accounting for utilization in 53 studies. Other statistical models that were used include difference in differences, path analysis model, principal components analysis, time series model, structural equation modelling and root-mean squared error.

Finally, some authors of the included articles discussed important elements that need to be considered when implementing and evaluating the impact of a BI intervention (*n* = 23), and some common themes emerged. Inaccurate reporting, such as underreporting of income streams among the treatment group and inability to recall past experiences, was the most common consideration highlighted by the authors (*n* = 9). High rate of attrition was also cited as a common issue that was encountered during longitudinal BI programs that lasted for several years (*n* = 7). The next common consideration was related to recruitment (*n* = 6). For example, a study examining the MPUCT program in India emphasized the importance of cluster-level randomization to mimic the universality of BI and to prevent distortion of BI effects that can be delivered through community-level mechanism [45]. Two studies underscored the need to select control and treatment populations with comparable characteristics to avoid inaccurate estimation of BI effects due to unaccounted confounding variables [38, 68]. Several researchers also cautioned against extrapolating the results of a single BI experiment to the national level, considering the differences in the population characteristics, local policies, and the existing welfare systems (*n* = 4) [38, 69–71]. In addition, some authors discussed the potential attenuation or exaggeration of the BI impacts due to the temporary nature of the BI experiments, which could influence participants to behave differently than if the intervention were longer or permanent (*n* = 3) [71–73]. Other important considerations that were discussed include the high cost of running a BI intervention, which can pose a challenge for replication [44, 74, 75]; the relatively rare instances of outcome of interests such as divorce that can influence the reliability of the estimates [38, 72, 75]; community influence on the acceptance of BI [76, 77]; and the importance of selecting an appropriate method for transferring the fund to accommodate participants who may not have access to a bank account [76].

## Discussion

In summary, 86 studies that spoke to 10 types of BI experiments were identified. The studies were diverse in nature and occurred within the past six decades. Our analysis revealed several consistent elements of the BI evaluation methods despite geographical differences. Most of the BI experiments implemented RCT design and randomized the sample selection at household or individual levels. Two BI experiments in rural settings, the MPUCT in India and the Give Directly Unconditional Cash Transfer Program in Kenya adopted cluster-randomized controlled trials, where randomizations occurred at the village-level. The advantage of this approach is its ability to mirror a universal basic income program where everybody receives an income and to capture any effects of basic income that are operated through community-level mechanisms and are facilitated by behavioural adaptation [45, 76]. However, this approach must take into account the higher degree of similarities among individuals within a cluster relative to those between different clusters.

The Namibian BIG Project and the Alaska Permanent Dividend Fund used observational design to evaluate the impact of BI. The BIG project was a pilot study aimed to move forward the nation’s discussion around basic income and therefore chose to observe only one village that was manageable in size and experienced a high degree of poverty [78]. In contrast, the Alaskan Permanent Dividend Fund is a state-funded program that has been running for almost 4 decades. The study examining this program utilized census and population-based survey data and chose difference-in-differences (DiD) analysis approach that allows for comparison between a group that is exposed to a treatment and another unexposed group, while taking into account differences that already exist prior to the treatment [42]. By employing this approach, researchers were able to both examine the evolution of the labour market in Alaska prior to and following the introduction of the dividend fund and compare it against the changes in labour market in other states that did not implement a similar program. Because of its large scale and the need to accumulate longitudinal data that can span over decades, this type of study is hard to replicate, particularly in a setting that does not have a reliable system to maintain population data. This approach also requires careful selection of other locations that are comparable in its population characteristics to serve as appropriate controls.

Various debates have emerged that discuss the effectiveness of study designs used in the evaluation of BI experiments. Widerquist [79] argues that due to their narrow focus and context, controlled, small-scale experiments have limited ability in uncovering the potential benefits of BI. The experiments included in this study involved relatively large numbers of participants and collected longitudinal data that spanned over several years and touched on various domains. The large sample size is important in producing reliable results with a high degree of precision and power, especially when differences between populations were assessed. Other researchers contend that conventional RCTs which account for the majority of experiments included in this review may not be an appropriate approach for demonstrating the effectiveness of BI. Scholars argue that the provision of income to a randomized cohort of individuals overlooks the influence of structural factors on BI-related outcomes [80]. These structural factors include social norms, role modeling, business responses, collective action and work for the underemployed [80]. In light of this, future BI experiments can consider other designs, such as clustered RCT or other longitudinal study designs that provide BI to all members of a specific cluster, such as a neighbourhood or a city [80, 81].

Overall, the studies placed a particular focus on labour-related outcomes, which included job searching attitudes and hours worked. Additional outcomes included impact on marital stability and dissolution, educational investments and outcomes, awareness of social services, level of debt, wage, and home ownership. A few studies (*n* = 11) analyzed the impact of BI on health-related outcomes as their main outcomes, including hospitalization. In many cases, the methods and outcomes were driven by the time period in which the BI experiments were conducted. In the 1960’s and 70’s, a strong focus was placed on the impact of BI on labour activities while more recent literature has incorporated a more holistic picture of BI’s impacts on factors such as social connectedness, health outcomes and education. The shift in focus towards health and education outcomes in studies dated after the 1990’s may be attributed to the societal recognition of the importance of the social determinants of health [7, 8, 13, 14].

Almost all studies used self-reported data, which was collected using surveys administered in person serially, complemented with administrative data. The common use of surveys in BI experiments was also noted in another review [27]. Several evidences have emerged on potential reporting bias due to underreporting of level of income and employment among the treatment group, particularly when the amount of benefits received is dependent on their income level [56, 82]. Recall bias is also a potential issue that researchers must contend with when using self-reported information. The SIME/DIME experiment, for example, asked participants to recall income information from the previous 4 months and more recent events may influence their perspectives and subsequent responses [83]. Therefore, data from alternative sources, such as unemployment insurance agencies or tax reports can be considered in future BI experiments to ensure accurate information. Appropriate training for interviewers and clear reporting forms are also potential measures to consider for reliable data collection [84].

When designing a BI experiment, researchers will need to consider the sample size of individuals who present their outcomes of interest. A study focusing on marital dissolution, for example, explained that their analytical approach must address the issue of small number of divorce occurrences to reliably measure the relationship between BI and divorce rates [72]. High rates of attrition, particularly in longitudinal studies spanning over a number of years, can also reduce the effective sample size and introduce bias if there are differential attrition rates between the control and the experimental groups, or if there is a fundamental difference in the characteristics of the participants who drop out and those who remain [74, 84]. Furthermore, future BI experiments could assess the dose-specific effects of income subsidy on the population, as was done during the NJ experiment that collected behaviour data from a sample that was given various amount of benefits [85].

The local resources to support the BI experiments will also need to be carefully considered, particularly when they are implemented in a rural area with limited access to technology. The MPUCT in India described difficulties in providing cash to participants as the majority of households did not have bank accounts, and physical transfer of funds proved to be challenging given the remote and hard to reach location of the experiment site [76]. Finally, existing presence of social agencies in the communities and local policies around social welfare may also influence the take-up rate of the BI intervention, and any attempt to compare experimental and control sites must consider these differences [76, 77].

This systematic review provides various implications for future research and intervention implementation related to BI. Primarily, this review has identified what previous evaluations of BI interventions have used as outcome measures when determining overall effectiveness. Identifying these outcomes not only provides a basis for future research, but also identifies existing gaps in knowledge with respect to the assessment of BI interventions (e.g., how does BI influence rates of violence?). Moving beyond the identification of common outcome measures, methods which have been incorporated in evaluations of BI interventions have been identified. This again provides future researchers or program implementers information on how to meaningfully evaluate a BI intervention. This systematic review demonstrates the range of impact BI interventions have on individuals, all of which contribute to overall health and well-being. However, the findings also outline common challenges that may be faced when assessing a BI intervention, including cost, which again can assist in developing pragmatic evaluation strategies.

This review has a number of strengths. It is the first systematic review to focus on the methodology used to evaluate BI in numerous experiments. All studies in this field from all countries were included and the similarities and differences on various aspects of their methodology were described. This review also has limitations. First, experiments with conditional cash transfers (e.g., Brazil’s *Bolsa Familia*, Italy’s *Sostegno per l’Inclusione Attiva*, and Uganda’s Youth Opportunities Programs) were not included. BI experiments that were still in the planning phases or had been prematurely cancelled (e.g., in Scotland, the Netherlands and Ontario, Canada) were excluded. Our study’s exclusion of qualitative methods of evaluation limited its insight on individual’s perceptions of BI. Furthermore, the inconsistency in the reporting of methods, which can be attributed to the wide array of included studies, poses a challenge in determining the effectiveness of specific methods.

## Conclusions

Income represents a key social determinant of health, which aids in the attainment of employment and maintenance of health and well-being [86]. The implementation of BI studies are particularly unique in that they are shaped by the policy priorities of the government in power. Research and interest in BI will continue to grow as states recognize that the current models of welfare are inadequate, overly bureaucratic and lead to further harms due to welfare-state conditions. The current social, health and political climate that has developed as a result of the COVID-19 pandemic, presents a unique opportunity to discuss the impacts of BI on overall health and well-being. This systematic review provides future researchers and program developers the tools needed to develop and evaluate BI as a method of social intervention effectively.

As identified by this review, there are various outcome measures and methods that have been implemented to evaluate existing BI interventions. Moving beyond the integration of RCTs, future research in this area should evaluate the rationale for different methodological approaches, while considering novel methods used to capture individual-level data, including through administrative data linkage. Additional methods that should be assessed in relation to BI intervention evaluations include the use of qualitative methods. Completing accurate and rigorous evaluations of social interventions, such as BI, will not only ensure that effective programmatic and policy-level changes occur, but will also aid in the improvement of the health and well-being of various populations.

## Supplementary Information

**Additional File 1.** Search Strategy and List of Searched Databases.

**Additional File 2.** Assessment of Quality of Individual Articles.

## Data Availability

Data sharing is not applicable to this article as no datasets were generated or analyzed during the current study.

## Abbreviations

BI: Basic Income
COVID-19: Coronavirus Disease of 2019
Gary: Gary Income Maintenance Experiment
IME: Income Maintenance Experiments
MPUCT: Madhya Pradesh Unconditional Cash Transfer Project
NIT: Negative Income Tax
NJ: New Jersey Income Maintenance Experiment
RCT: Randomized Controlled Trial
RIME: Rural Income Maintenance Experiment
SIME/DIME: Seattle-Denver Income Maintenance Experiment
UCT: Unconditional Cash Transfers
US: United States
