# Supplementary material for: Exploring different methods to evaluate the impact of basic income interventions: a systematic review
Source: Int J Equity Health. 2021 Jun 16;20:142. doi: 10.1186/s12939-021-01479-2 (PMC8206888; doi:10.1186/s12939-021-01479-2)
Supplement: Supplementary file 1 — Additional File 1. Search Strategy and List of Searched Databases. [file 12939_2021_1479_MOESM1_ESM.docx]

# **Additional File 1: Search Strategy and List of Searched Databases**

The following search strategy was carried out in the databases listed below, with no limits used:

"basic income" OR "guaranteed annual income" OR "guaranteed minimum income" OR "minimum income" OR "negative income tax" OR "optimal income transfer*" OR mincome OR demogrant* or "citizen* income" OR "universal income" or "y combinator".

Peer-reviewed and grey articles were searched from 24 databases listed below:

- Scopus
- OVID databases searched simultaneously:
  - Medline
  - PsycINFO
  - Embase
  - Social Work Abstracts
- OVID Cochrane Database of Systematic Reviews & Cochrane Central Register of Controlled Trials
- CINAHL (EBSCOhost)
- Web of Science databases searched simultaneously:
  - Science Citation Index Expanded
  - Social Sciences Citation Index
  - Arts & Humanities Citation Index
  - Conference Proceedings Citation Index- Science
  - Conference Proceedings Citation Index- Social Science & Humanities
  - Emerging Sources Citation Index (ESCI)
- EBSCOhost databases searched simultaneously:
  - Alternative Press Index
  - Business Source Premier
  - Left Index
- ProQuest databases searched simultaneously:
  - Applied Social Sciences Index & Abstracts (ASSIA)
  - Canadian Research Index
  - International Bibliography of the Social Sciences (IBSS)
  - PAIS Index
  - ProQuest Dissertations & Theses Global
  - Sociological Abstracts
  - Worldwide Political Science Abstracts
