# Supplementary material for: Exploring different methods to evaluate the impact of basic income interventions: a systematic review
Source: Int J Equity Health. 2021 Jun 16;20:142. doi: 10.1186/s12939-021-01479-2 (PMC8206888; doi:10.1186/s12939-021-01479-2)
Supplement: Supplementary file 2 — Additional File 2. Assessment of Quality of Individual Articles. [file 12939_2021_1479_MOESM2_ESM.docx]

# **Additional File 2: Assessment of Quality of Individual Articles**

1. **Articles appraised with JBI checklist for RCTs** (35)

List of Questions:

1. Was true randomization used for assignment of participants to treatment groups?
2. Was allocation to treatment groups concealed?
3. Were treatment groups similar at the baseline?
4. Were participants blind to treatment assignment?
5. Were those delivering treatment blind to treatment assignment?
6. Were outcomes assessors blind to treatment assignment?
7. Were treatment groups treated identically other than the intervention of interest?
8. Was follow up complete and if not, were differences between groups in terms of their follow up adequately described and analyzed?
9. Were participants analyzed in the groups to which they were randomized?
10. Were outcomes measured in the same way for treatment groups?
11. Were outcomes measured in a reliable way?
12. Was appropriate statistical analysis used?
13. Was the trial design appropriate, and any deviations from the standard RCT design (individual randomization, parallel groups) accounted for in the conduct and analysis of the trial?

Results:

| **Authors, Years** | **Q1** | **Q2**^a^ | **Q3** | **Q4**^a^ | **Q5**^a^ | **Q6**^a^ | **Q7** | **Q8** | **Q9** | **Q10** | **Q11** | **Q12** | **Q13** | **Total Number of “Yes”** |
| --- | --- | --- | --- | --- | --- | --- | --- | --- | --- | --- | --- | --- | --- | --- |
| Beck et al, 2015 (52) | Yes | N/A | Yes | N/A | N/A | N/A | Yes | Yes | Yes | Yes | Yes | Yes | Yes | 9 |
| SEWA Bharat, 2014 (53) | Yes | N/A | Unclear | N/A | N/A | N/A | Unclear | Yes | Yes | Yes | Yes | Yes | Yes | 7 |
| Cain and Wissoker, 1990 (72) | No | N/A | Yes | N/A | N/A | N/A | Yes | Yes | Yes | Yes | Yes | Yes | Yes | 8 |
| Cain et al, 1974 (92) | No | N/A | Yes | N/A | N/A | N/A | Yes | Yes | Yes | Yes | Yes | Yes | Yes | 8 |
| Calnitsky, 2016 (55) | Yes | N/A | Yes | N/A | N/A | N/A | Yes | Yes | Yes | Yes | Yes | N/A | Yes | 8 |
| Choudhry and Hum, 1995 (94) | Yes | N/A | Yes | N/A | N/A | N/A | Yes | Yes | Yes | Yes | Yes | Yes | Yes | 9 |
| Curry, 1981 (95) | No | N/A | Yes | N/A | N/A | N/A | Yes | Yes | Yes | Yes | Yes | Yes | Yes | 8 |
| Elesh and Lefcowitz, 1977 (96) | No | N/A | Unclear | N/A | N/A | N/A | Yes | Yes | Yes | Yes | Yes | Yes | Yes | 7 |
| Forget, 2011 (10) | Yes | N/A | Yes | N/A | N/A | N/A | Unclear | Yes | Yes | Yes | Yes | Yes | Yes | 8 |
| Forget, 2013 (64) | Yes | N/A | Yes | N/A | N/A | N/A | Unclear | Yes | Yes | Yes | Yes | Yes | Yes | 8 |
| Greenberg and Halsey, 1983 (56) | No | N/A | Unclear | N/A | N/A | N/A | Yes | Unclear | Yes | Yes | Yes | Yes | Yes | 6 |
| Greenberg, Moffitt, Friedmann, 1981 (65) | No | N/A | Unclear | N/A | N/A | N/A | Yes | Yes | Yes | Yes | Yes | Yes | Yes | 7 |
| Haushofer and Shapiro, 2013 (36) | Yes | N/A | Yes | N/A | N/A | N/A | Yes | Yes | Yes | Yes | Yes | Yes | Yes | 9 |
| Heffernan, 1977 (60) | No | N/A | Unclear | N/A | N/A | N/A | Yes | Unclear | Yes | Yes | Yes | Yes | Yes | 6 |
| Hum and Choudry, 1992 (100) | Yes | N/A | Unclear | N/A | N/A | N/A | Yes | Unclear | Yes | Yes | Yes | Yes | Yes | 7 |
| Hum and Simpson, 1991 (101) | Yes | N/A | Yes | N/A | N/A | N/A | Yes | Unclear | Yes | Yes | Yes | Yes | Yes | 8 |
| Kaluzny, 1979 (47) | Unclear | N/A | Unclear | N/A | N/A | N/A | Yes | Yes | Yes | Yes | Yes | Yes | Yes | 7 |
| Kangas et al, 2019 (104) | Yes | N/A | No | N/A | N/A | N/A | Yes | Yes | Yes | Yes | Yes | Yes | Yes | 8 |
| Kehrer and Wolin, 1979 (63) | No | N/A | Yes | N/A | N/A | N/A | Yes | Yes | Yes | Yes | Yes | Yes | Yes | 8 |
| Kershaw, 1972 (111) | No | N/A | Yes | N/A | N/A | N/A | Yes | Yes | Yes | Yes | Yes | Yes | Yes | 8 |
| McDonald and Stephenson, 1979 (114) | No | N/A | Yes | N/A | N/A | N/A | Yes | Yes | Yes | Yes | Yes | Yes | Yes | 8 |
| Murray and Pateman, 2012 (67) | Yes | N/A | Yes | N/A | N/A | N/A | Yes | Yes | Yes | Yes | Yes | Unclear | Yes | 8 |
| Nicholson and Wright 1977 (37) | No | N/A | Unclear | N/A | N/A | N/A | Yes | Yes | Yes | Yes | Yes | Yes | Yes | 7 |
| O'Connor and Madden, 1979 (117) | No | N/A | Yes | N/A | N/A | N/A | Yes | Yes | Yes | Yes | Yes | Yes | Yes | 8 |
| Robins, 1980 (38) | No | N/A | Unclear | N/A | N/A | N/A | Yes | Yes | Yes | Yes | Yes | Yes | Yes | 7 |
| Standing, 2015 (122) | Yes | N/A | Yes | N/A | N/A | N/A | Yes | Yes | Yes | Yes | Yes | N/A | Yes | 8 |
| Wright, 1975 (39) | No | N/A | Yes | N/A | N/A | N/A | Yes | Yes | Yes | Yes | Yes | Yes | Yes | 8 |

^a^The nature of basic income intervention, where participants will always know whether they receive the income or not, does not permit blinding or concealment. As such, blinding procedure is not applicable to research on basic income intervention.

1. **Articles appraised with JBI checklist for economic evaluations** (35)

List of questions:

1. Is there a well-defined question?
2. Is there comprehensive description of alternatives?
3. Are all important and relevant costs and outcomes for each alternative identified?
4. Has clinical effectiveness been established?
5. Are costs and outcomes measured accurately?
6. Are costs and outcomes valued credibly?
7. Are costs and outcomes adjusted for differential timing?
8. Is there an incremental analysis of costs and consequences?
9. Were sensitivity analyses conducted to investigate uncertainty in estimates of cost or consequences?
10. Do study results include all issues of concern to users?
11. Are the results generalizable to the setting of interest in the review?

Results:

| **Authors, Years** | **Q1** | **Q2** | **Q3** | **Q4** | **Q5** | **Q6** | **Q7** | **Q8** | **Q9** | **Q10** | **Q11** | **Total Number of “Yes”** |
| --- | --- | --- | --- | --- | --- | --- | --- | --- | --- | --- | --- | --- |
| Ashenfelter, 1990 (74) | Yes | Yes | No | N/A | Yes | Yes | Yes | No | No | Yes | Yes | 7 |
| Burtless and Hausman, 1978 (90) | Yes | Yes | Yes | N/A | Yes | Yes | Yes | Yes | No | Yes | Yes | 9 |
| Groeneveld et al, 1980 (70) | Yes | Yes | Yes | N/A | Yes | Yes | Yes | Yes | Yes | Yes | Yes | 10 |
| Haushofer and Shapiro, 2013 (36) | Yes | No | No | N/A | Yes | Yes | Yes | No | No | Yes | Yes | 6 |
| Hollister, 1974 (99) | No | Yes | Yes | N/A | Yes | Yes | Yes | No | No | Yes | Yes | 7 |
| Hum and Simpson, 1993 (102) | Yes | Yes | Yes | N/A | Yes | Yes | Yes | No | Yes | Yes | Yes | 9 |
| Johnson, 1980 (61) | Yes | No | No | N/A | Yes | Yes | Yes | No | No | Yes | Yes | 6 |
| Keeley et al, 1978 (106) | Yes | Yes | Yes | N/A | Yes | Yes | Yes | No | No | Yes | Yes | 8 |
| Keeley and Robins, 1979 (107) | Yes | No | No | N/A | Yes | Yes | No | No | No | Yes | Yes | 5 |
| Keeley, 1980 (58) | Yes | No | No | N/A | Yes | Yes | No | No | No | Yes | Yes | 5 |
| Keeley, 1980 (108) | Yes | No | No | N/A | Yes | Yes | Yes | No | No | Yes | Yes | 6 |
| Keeley, 1980 (59) | Yes | No | Yes | N/A | Yes | Yes | No | No | No | Yes | Yes | 6 |
| Keeley, 1987 (109) | Yes | Yes | Yes | N/A | Unclear | Yes | Yes | No | No | Yes | Yes | 7 |
| Kerachsky, 1977 (110) | Yes | No | Unclear | N/A | Unclear | Unclear | No | No | No | Yes | Yes | 3 |
| Maynard, 1977 (66) | Yes | No | Yes | N/A | Yes | Yes | No | No | No | Yes | Yes | 6 |
| Maynard and Murnane, 1979 (113) | Yes | No | Unclear | N/A | Yes | Yes | No | Yes | No | Yes | Yes | 6 |
| Moffitt, 1979 (115) | Yes | No | Unclear | N/A | Unclear | Unclear | No | No | No | Yes | Yes | 3 |
| Nicholson and Wright 1977 (37) | Yes | Yes | Unclear | N/A | Yes | Yes | No | Yes | Yes | Yes | Yes | 8 |
| Robins, 1980 (38) | Yes | Yes | Yes | N/A | Yes | Yes | No | Yes | Yes | Yes | Yes | 9 |
| Robins, Tuma, and Yaeger, 1980 (119) | Yes | Yes | Yes | N/A | Yes | Yes | Yes | Yes | Yes | Yes | Yes | 10 |
| Robins and West, 1986 (120) | Yes | Yes | Yes | N/A | Yes | Yes | Yes | Yes | Yes | Yes | Yes | 10 |
| Ross, 1970 (121) | Yes | Yes | Yes | N/A | Yes | Yes | Yes | Yes | Yes | Yes | Yes | 10 |
| Stephens, 2007 (123) | Yes | Yes | Yes | N/A | Yes | Yes | Yes | Unclear | Yes | Yes | Yes | 9 |
| Weiss, Hall, and Dong, 1980 (125) | Yes | Yes | Yes | N/A | Yes | Yes | Yes | Unclear | Unclear | Yes | Yes | 8 |
| West, 1980 (126) | Yes | Yes | Yes | N/A | Yes | Yes | Yes | No | Yes | Yes | Yes | 9 |
| West, 1980 (127) | Yes | Yes | Yes | N/A | Yes | Yes | Yes | Yes | No | Yes | Yes | 9 |
| Wright, 1975 (39) | Yes | Yes | Yes | N/A | Yes | Yes | Yes | Yes | Yes | Yes | Yes | 10 |

1. **Articles appraised with JBI checklist for case series** (35)

List of questions:

1. Were there clear criteria for inclusion in the case series?
2. Was the condition measured in a standard, reliable way for all participants included in the case series?
3. Were valid methods used for identification of the condition for all participants included in the case series?
4. Did the case series have consecutive inclusion of participants?
5. Did the case series have complete inclusion of participants?
6. Was there clear reporting of the demographics of the participants in the study?
7. Was there clear reporting of clinical information of the participants?
8. Were the outcomes or follow up results of cases clearly reported?
9. Was there clear reporting of the presenting site(s)/clinic(s) demographic information?
10. Was statistical analysis appropriate?

Results:

| **Authors, Years** | **Q1** | **Q2** | **Q3** | **Q4** | **Q5** | **Q6** | **Q7** | **Q8** | **Q9** | **Q10** | **Total Number of “Yes”** |
| --- | --- | --- | --- | --- | --- | --- | --- | --- | --- | --- | --- |
| Haarmann, 2008 (40) | Unclear | Unclear | No | Yes | Yes | Yes | N/A | Yes | Yes | Yes | 6 |
| Haarmann et al, 2009 (41) | Unclear | Unclear | No | Yes | Yes | Yes | N/A | Yes | Yes | Yes | 6 |

1. **Article appraised with JBI checklist for quasi-experimental studies** (35)

List of questions:

1. Is it clear in the study what is the ‘cause’ and what is the ‘effect’ (i.e., there is no confusion about which variable comes first)?
2. Were the participants included in any comparisons similar?
3. Were the participants included in any comparisons receiving similar treatment/care, other than the exposure or intervention of interest?
4. Was there a control group?
5. Were there multiple measurements of the outcome both pre and post the intervention/exposure?
6. Was follow up complete and if not, were differences between groups in terms of their follow up adequately described and analyzed?
7. Were the outcomes of participants included in any comparisons measured in the same way?
8. Were outcomes measured in a reliable way?
9. Was appropriate statistical analysis used?

Results:

| **Authors, Years** | **Q1** | **Q2** | **Q3** | **Q4** | **Q5** | **Q6** | **Q7** | **Q8** | **Q9** | **Total Number of “Yes”** |
| --- | --- | --- | --- | --- | --- | --- | --- | --- | --- | --- |
| Jones and Marinescu, 2018 (42) | Yes | Yes | Unclear | Yes | Yes | Yes | Yes | Yes | Yes | 8 |
